# Care Plan Writing in Nursing Education: Challenges, Competence, and Clinical Preparedness

**DOI:** 10.3390/nursrep15040134

**Published:** 2025-04-16

**Authors:** Florence Mei Fung Wong

**Affiliations:** School of Nursing, Tung Wah College, Hong Kong SAR, China; florencewong@twc.edu.hk; Tel.: +852-3468-6838

**Keywords:** nursing care plans, undergraduate nursing students, learning attitudes, clinical competence, nursing education

## Abstract

**Background**: Care plans are a critical tool in nursing education because they enhance clinical competence; however, undergraduate students often face challenges in writing them effectively, which can impact their readiness for clinical practice. While existing research predominantly focuses on care plans within specific clinical contexts, little is known about how students experience the learning process and how these experiences shape their professional development. **Objectives**: This study aimed to explore the experiences of undergraduate nursing students in writing care plans to understand the impact on their clinical competence and identify strategies for improvement. **Design**: A qualitative phenomenological study utilizing focus group interviews was conducted. **Methods:** Semi-structured interviews with open-ended questions were conducted with 15 undergraduate nursing students in six focus groups. Data were analyzed using Colaizzi’s method to identify key themes. **Results:** Four main themes emerged: (1) enhancement and integration of knowledge and skills, (2) initiative learning and motivation, (3) adequate support and feedback from tutors, and (4) difficulties in transitioning from classroom learning to clinical practice. The findings highlight that care plan writing enhances students’ competence in patient care, with positive learning attitudes and tutor feedback playing crucial roles. However, students encounter difficulties in applying theoretical knowledge to complex clinical scenarios, particularly in prioritizing interventions and managing time effectively. **Conclusions:** Writing care plans not only fosters personal and professional development but also enhances students’ clinical competence, preparing them for real-world practice. Nurse tutors are encouraged to promote consistent practice in care plan writing, provide timely feedback, and share clinical experiences to support students’ learning. These findings underscore the need to reframe care plans as developmental tools rather than mere tasks for clinical transition, ultimately enhancing the quality of patient care.

## 1. Introduction

The increasing complexity of healthcare services demands that nursing education equips students with a comprehensive blend of both theoretical knowledge and practical skills to deliver safe, high-quality patient care [1,2]. At the core of nursing education lies the development of clinical competence, which enables students to effectively integrate and apply their learning to meet the unique health needs of individual patients. One essential tool for fostering this competence is the nursing care plan, which serves as a structured guide for delivering individualized, evidence-based, and holistic care [3,4].

Ensuring nursing competence in the integration of knowledge and skills is crucial for optimal patient care. Various pedagogical approaches, such as concept maps [5], the nursing process [2,3], small group discussions [6], and specific teaching tools [7], are utilized to facilitate student learning and enhance their understanding of specific topics. Care plans serve not only as a vital teaching tool but also as a practical framework that enhances students’ patient care-related competence [3,8]. By systematically identifying patient problems, planning interventions, and evaluating outcomes, care plans improve interdisciplinary communication among healthcare teams, ensure continuity of care across shifts, and promote cost-effective, ethical, and safe patient care in current clinical settings [2,3]. Aligned with the nursing process, care plans guide students in applying evidence-based knowledge, refining their critical thinking, clinical judgment, and professional autonomy [9,10,11]. As such, writing care plans is a key learning activity that bridges theory and practice, preparing students for the complexities of clinical environments [10,11].

Despite their recognized value, existing research has predominantly focused on specific patient populations or advanced treatments in particular clinical contexts, such as chronic illness management or post-operative care, with limited focus on the broader educational experiences of undergraduate nursing students [12,13]. Many students encounter challenges in writing effective care plans, particularly for complex cases, often heavily relying on external resources such as peer discussions and online materials. While studies acknowledge the role of care plans in professional and clinical competence development, there remains a critical gap in understanding how students experience and learn from the process of care plan writing as an educational tool, and their experiences shape their clinical competence and professional development, as care plan writing directly fosters students’ ability to internalize care planning, impacting their transition to practice.

This study aimed to address these gaps by exploring the lived experiences of undergraduate nursing students in writing care plans through a phenomenological approach with focus group interviews. By examining how care plan writing impacts students’ learning, clinical competence, and professional development, this research aims to explore the nuances of undergraduate nursing students’ experiences in writing care plans, focusing on their learning processes, challenges, and the impact on their clinical competence and professional development. This qualitative research provides evidence-based insights to identify students’ subjective challenges and how these challenges shape their professional identity development, empowering nurse educators to refine teaching strategies and transform care plan writing from a routine exercise into a meaningful bridge between classroom and clinical practice, ultimately enhancing nursing education and patient care outcomes.

### Research Aims and Objectives

This study aimed to explore undergraduate nursing students’ lived experiences in writing care plans. The objectives were to identify their perceived challenges and learning processes and understand how actionable strategies to improve care plan pedagogy can effectively bridge the gap between classroom learning and clinical practice.

## 2. Materials and Methods

### 2.1. Design

A qualitative study using semi-structured focus group interviews was conducted to explore undergraduate nursing students’ experiences in writing care plans. This design facilitated an in-depth exploration of participants’ perspectives and allowed researchers to follow up on emerging themes during the interviews. A phenomenological approach is uniquely suited to uncover these nuances, as it prioritizes students’ lived experiences, revealing not just what they learn but how they ascribe meaning to the process.

The Consolidated Criteria for Reporting Qualitative Research (COREQ-32) [14] (see Table S1) guided the study process to ensure that essential components are reported with sufficient information to improve the quality and transparency of the qualitative research.

### 2.2. Participants and Setting

Purposive sampling was used to recruit undergraduate nursing students from a professional training institution who had experience in writing care plans between 2 January and 31 March 2018. Eligible participants were aged 18 or older, enrolled in a nursing program, and selected to ensure diversity based on their year of study and clinical exposure.

### 2.3. Data Collection

Ethical approval was obtained from the institution’s Research Ethics Committee, and the study adhered to the principles of the Declaration of Helsinki. Participants were provided with an information sheet detailing the study’s purpose, their involvement, potential risks and benefits, data usage, and consent to publish anonymized data. Written informed consent was obtained, and participants completed a demographic questionnaire prior to the interviews.

Face-to-face focus group interviews, consisting of three to five participants each, were conducted by the principal investigator (PI) in a quiet room at the institution. Interviews were conducted in Cantonese or English, based on participants’ preferences. The PI began with ice-breaker questions to ease participation stress, followed by a semi-structured interview guide (Supplementary S1). The opening question, “Share with me your experience of writing care plans during your study”, was supplemented with probe and prompt questions to clarify and expand responses. Each interview lasted 45 to 90 min and was audio-recorded. A research assistant (RA) took field notes, and the PI observed participants’ behaviors and reactions. Data collection continued until saturation was achieved, with no new themes emerging.

### 2.4. Data Analysis

This study employed Colaizzi’s [15] seven-step phenomenological method to systematically analyze students’ lived experiences with care plan writing. This approach was selected for its capacity to uncover the nuanced, subjective dimensions of learning processes while upholding methodological rigor. The analysis began with verbatim transcription of all the audio recordings by the RA, cross-verified against the original recordings by the PI for accuracy. The PI and RA immersed themselves in the data through repeated readings of transcripts and annotated initial impressions in a reflective journal to establish familiarity with the dataset before formal coding commenced.

In the subsequent phase, the team independently identified significant statements—direct participant quotations capturing essential aspects of care plan writing in the original context. These statements were then interpreted to derive meanings. The analysis progressed through iterative clustering, where related meanings were grouped into emergent categories. Through constant comparative analysis, these categories were synthesized into overarching themes that encapsulated the multidimensional nature of students’ experiences through deliberative dialogue.

To enhance trustworthiness, participants (40% of the sample) were contacted for member checking to validate the findings. Their feedback led to refinements in theme articulation. The final synthesis articulated the fundamental structure of the phenomenon; care plan writing emerged as both a developmental gateway and a source of tension, where students navigated between idealized protocols and authentic clinical complexity. This comprehensive analytical approach ensured findings remained deeply grounded in participants’ actual experiences while providing actionable insights for nursing education.

### 2.5. Study Rigor and Triangulation

Study rigor and trustworthiness were ensured through purposive sampling to enable comparisons across different student cohorts. Triangulation was achieved through persistent observation, field notes, and independent coding by the PI and RA, followed by cross-verification to minimize bias. Member checking was conducted to validate the findings, allowing participants to clarify or refine their statements and themes [16]. These measures ensured the production of robust and dependable findings.

## 3. Ethical Considerations

The study adhered to the Declaration of Helsinki and received ethical approval from the institution’s Research Ethics Committee (NUR/SRC/20171220/007). Participants provided informed consent, and all personal data were anonymized to ensure confidentiality and protect participants’ privacy and rights throughout the research process.

## 4. Results

The study included 15 undergraduate nursing students (eight males, seven females) aged 20–25 years, with a mean age of 22.46 (SD1.5) years, enrolled in years three to five of a bachelor’s degree program. Participants had 2–5 years of experience in writing care plans. Table 1 shows the demographic characteristics.

Analysis of their experiences revealed four main themes: (1) enhancement and integration of knowledge and skills, (2) initiative learning attitudes and motivation, (3) adequate support and feedback from tutors, and (4) difficulties in transitioning from classroom learning to clinical practice. Table S2 illustrates a coding tree, and Figure S1 shows the identified themes and subthemes.

### 4.1. Theme 1: Enhancement and Integration of Knowledge and Skills

#### 4.1.1. Subtheme 1: Utilization of Evidence-Based Knowledge and Skills

All student participants emphasized that writing care plans deepened their understanding and application of evidence-based knowledge and skills. They recognized the importance of individualized care, as each patient has unique health needs. One student described care plan writing as a tool for designing and implementing more appropriate and effective patient care: *“When we have a case designed by our tutor, we identify the patient’s needs and write a care plan. We apply evidence-based knowledge and practical skills, like pathophysiology and pharmacology, to resolve patient problems.”* (Student I)

Another participant highlighted the importance of self-directed learning to strengthen knowledge: *“We need to understand the topic well. If we don’t, we do self-study—reading textbooks, searching online, or consulting instructors. There’s a lot of information, so we screen interventions to fit the patient’s unique needs. Each patient is different, so we tailor interventions based on their specific problems.”* (Student W)

Some students expressed that their experience of clinical practice also enhanced their ability to write care plans. A student participant gave the following description: *“In clinical practicum, I encountered patients with conditions similar to our case scenarios. The care plans we wrote in class weren’t always applicable, but caring for real patients taught me more, further enhancing my competence.”* (Student N)

#### 4.1.2. Subtheme 2: Advanced Skill Development

Most student participants described care plan writing as a valuable tool for developing advanced skills, such as critical thinking and problem-solving. One student explained how this process enhanced her abilities: *“Writing a care plan involves critically analyzing the case and making decisions. Each patient has unique needs, so this process trains us to think deeply and provide appropriate care. This training is essential and makes us more competent in patient care.”* (Student S)

Another participant elaborated on how care plan writing fosters critical thinking: *“Critical thinking is definitely developed through writing care plans. We integrate all the knowledge and skills we’ve learned, not just from one course but from our entire study. For example, with a patient in pain, we consider both basic pain relief interventions and disease-specific measures. This decision-making process is complex and requires us to consider multiple factors, making care plan writing a crucial part of our training.”* (Student W)

A third student participant highlighted the importance of prioritization and organization: *“We learn to prioritize patient problems. A patient may have many issues, but we need to identify the most critical ones. With more practice and clinical experience, we become better at organizing problems and planning appropriate interventions.”* (Student S)

#### 4.1.3. Subtheme 3: Benefits to Patient Care

Most participants found care plans highly useful in clinical practice. After writing care plans for specific cases designed by their tutors, they were better able to identify patient problems and develop appropriate interventions. One student shared how care plans improved her clinical responsiveness: *“Care plans help me respond quickly in clinical practice. For example, with a patient experiencing sputum, I immediately prop them up, teach breathing exercises, and provide other interventions outlined in the care plan. This ensures we apply our knowledge and skills effectively.”* (Student K)

Another student participant emphasized the importance of understanding intervention rationales behind the provision of appropriate nursing care and the development of analytic skills and critical thinking: *“The challenge is thinking through the rationales behind interventions. Writing a care plan isn’t just listing actions—it’s about understanding why we do them. This process trains us to think critically and apply evidence-based practice, which is crucial when explaining care to patients.”* (Student B)

A third student participant highlighted how care plans boosted her confidence and competence in clinical practice through providing evidenced-based practice and applying an effective approach: *“Care plans provide a step-by-step approach to patient care, from assessment to implementation. They help me deliver effective care without mistakes, leading to better patient recovery. This builds my confidence, and my patients trust my care more.”* (Student K)

### 4.2. Theme 2: Initiative Learning Attitudes and Motivation

Most student participants acknowledged that writing care plans was a learning process, with competence enhancement depending on their attitudes and motivation. A junior student described her initial challenges and growth: *“Care plan writing was new to me when I started the program. At first, it was difficult, but with self-practice repeatedly, it became an achievement, especially when caring for patients in clinical practice.”* (Student P)

Another student participant emphasized the role of self-motivation: *“Self-motivation is key. Once we realize the importance of care plans, we push ourselves to learn more and improve.”* (Student A)

A third student shared a practical motivator: *“Care plans are part of our exams, so we’re motivated to learn and perform well.”* (Student N, laughter)

### 4.3. Theme 3: Adequate Support and Feedback from Tutors

#### 4.3.1. Subtheme 1: Timely Feedback from Tutors

All student participants emphasized the need for more support from their tutors, as care plans are a required part of course assessments. Many highlighted that timely feedback was crucial for improving their care plan writing. One student shared how feedback helped her improve: *“Practicing is important, but tutor feedback is essential. Sometimes, I thought my care plan was well-written, but the grade didn’t reflect that. Seeking feedback helps identify weaknesses and improve.”* (Student W)

Another student participant stressed the importance of constructive comments: *“Tutors should provide feedback after marking our care plans, so we know what mistakes we made and how to improve. Otherwise, we’re left guessing.”* (Student K, nods from others)

#### 4.3.2. Subtheme 2: Sharing of Clinical Experience by Tutors

Some student participants noted that while they often relied on textbooks and online resources to understand patient care, they found tutors’ clinical stories more impactful. These stories helped them better memorize interventions and rationales, enhancing their ability to write care plans. One student explained how tutors’ experiences enriched their learning: *“Tutors should share more clinical experiences to help us write care plans. Classroom learning lacks real patients, which limits our understanding. When tutors share their stories, it’s like creating mental pictures of interventions. For example, with a patient experiencing shortness of breath, our tutor explained how to prop up the patient, administer oxygen, assess, and provide further interventions until the problem is resolved. This kind of learning is far more valuable and effective.”* (Student N)

#### 4.3.3. Subtheme 3: Learning in a Group

Some student participants suggested that group work made care plan writing more efficient and effective. They explained that collaborating allowed them to leverage each other’s strengths and address weaknesses. One student shared her perspective: *“A care plan has many components, so working in a group helps us learn from each other. Some students excel at identifying problems, while others are better at writing interventions. By working together, we understand our strengths and weaknesses, which helps us improve. Sometimes, we don’t even realize our care plan isn’t well-written until we discuss it with others.”* (Student S, laughter from the group)

### 4.4. Theme 4: Difficulties in Transitioning from Classroom Learning to Clinical Practice

#### 4.4.1. Subtheme 1: Inadequate Time/Heavy Workload

Although care plans are used in clinical settings, participants found it challenging to write them during busy shifts. Those with clinical experience shared their struggles: *“Writing care plans in clinical practice is difficult. During my placement in a female medical unit, I couldn’t find time to write a care plan because bedside care took up my entire shift. Even when I tried, I couldn’t focus because I was constantly interrupted.”* (Student W)

#### 4.4.2. Subtheme 2: Handling More Complicated Cases in Clinical Practice

Most participants noted that classroom learning, which relies on case scenarios, often focuses on specific diseases or topics. However, they found it challenging to apply this knowledge to real patients in clinical practice, who often present with multiple, complex conditions. One student shared his perspective: *“Classroom scenarios are designed for learning specific topics, so the care plans we write don’t fully reflect the complexities of real patients. In clinical practice, patients often have multiple issues due to different diseases, making it difficult to prioritize problems and apply interventions. I’m still learning how to handle these complexities, so writing care plans for real patients feels overwhelming. Classroom learning provides a foundation, but in practice, care plans must be comprehensive and tailored to each patient’s unique needs. As a result, the interventions we practice in class may not always apply directly to real-world situations.”* (Student A)

## 5. Discussion

This qualitative study explored nursing students’ experiences with care plan writing using phenomenological analysis and identified four interconnected themes: (1) enhancement and integration of knowledge and skills, (2) initiative learning attitudes and motivation, (3) adequate support and feedback from tutors, and (4) difficulties in transitioning from classroom learning to clinical practice. These themes were further elaborated through subthemes, which provided deeper insights into the students’ experiences. For the first theme, subthemes included the utilization of evidence-based knowledge and skills, advanced skill development, and benefits to patient care. The second theme of adequate support and feedback from tutors encompassed timely feedback from tutors, the sharing of clinical experience by tutors, and learning in a group. Difficulties in transitioning to clinical practice were highlighted through subthemes focusing on inadequate time/heavy workload and handling more complicated cases in clinical practice. While the theme of initiative learning attitudes and motivation did not have specific subthemes, together, these themes shed light on both the successes and challenges students face in learning care plan writing, contributing to their personal and professional development [8,17,18]. The identified themes illuminate both the educational value and implementation challenges of this pedagogical tool. Table 2 summarizes the identified themes and subthemes regarding students’ learning experience in care plan writing.

The findings demonstrate that care plan development serves as a crucial bridge between theoretical knowledge and clinical practice while also identifying areas for potential improvement in current teaching methodologies. The process of crafting a care plan follows a systematic, step-by-step approach, identifying patient problems through comprehensive assessment, developing evidence-based interventions, and evaluating goal achievement [3,19]. Utilizing evidence-based knowledge ensures that care plans are not only safe and cost-effective but also promote patient recovery. Apart from its educational value, a care plan serves as a documentation guide for addressing patient needs and managing risks associated with specific health conditions. Care plans also facilitate effective communication among healthcare professionals, patients, and families, ensuring continuity of care, safety, and high-quality outcomes [8].

A nursing care plan showcases a student’s competence in understanding and addressing patient needs through appropriate interventions [19,20,21]. By applying classroom knowledge to clinical practice, students develop individualized care plans [20,21]. Therefore, nursing care plans serve as both an assessment tool and developmental exercise, demonstrating students’ growing competence in patient care planning and nurturing essential cognitive and behavioral skills crucial for delivering holistic, patient-centered care [19,20,21]. Our study with 15 participants confirms the value of care plans in translating classroom knowledge to clinical practice, developing comprehensive patient care understanding [10,11], and cultivating essential skills for holistic care. Participants emphasized how this structured approach helped them integrate classroom knowledge with hands-on experience, particularly when working with real patients in clinical settings. Competency progresses significantly across academic years, with senior students benefiting from repeated practice through assignments, projects, and authentic clinical experiences with real patients. Consequently, senior students are expected to demonstrate more sophisticated care planning abilities, indicating that repeated practice and accumulated clinical experience significantly contribute to competency development.

A valid and comprehensive care plan should be patient-centered, individualized, and rooted in evidence-based knowledge and practical steps [22,23]. When students develop care plans for individual patients, they apply existing knowledge and explore new information through various learning channels, such as self-study, consultations with tutors or mentors, group work, and online research [6,24]. This process requires students to critically evaluate the relevance and reliability of the information they gather. Participants in this study emphasized the importance of taking the initiative to seek additional knowledge, as it not only enriches their care plans but also fosters advanced skills like critical thinking and problem-solving [25,26]. Self-directed learning, motivated by intrinsic drive, is essential for deepening understanding and expanding competence [12,18].

Competence is cultivated through consistent practice and the application of evidence-based knowledge and skills. Through regularly writing care plans, students bridge the gap between classroom learning and real-world clinical practice, thereby improving the quality of care they deliver [23,27]. While scenario-based patients serve as valuable tools, offering a foundation for understanding specific topics, actual clinical practice requires a more nuanced and comprehensive approach.

However, care plans developed for scenario-based patients often fail to reflect the multifaceted issues encountered in real clinical practice. Classroom cases are typically straightforward and structured by tutors, whereas real patients frequently present with complex, overlapping health problems stemming from multiple diseases. Crafting care plans for such patients can be challenging, requiring advanced competence to effectively prioritize and address diverse issues. Continuous learning is essential to improving competency in care plan development. Although classroom learning provides a foundational understanding of care plan writing for specific conditions, clinical practice demands comprehensive, tailored care plans that cater to the unique needs of individual patients. As a result, interventions learned in the classroom may not always be directly translated to real-world clinical settings.

A care plan is a multifunctional tool that guides appropriate interventions to achieve patient outcomes based on their health needs. It not only supports nurses’ personal and professional development through self-directed learning but also enhances healthcare competence by integrating knowledge and skills into practice. Writing care plans helps students in prioritizing patient problems and designing appropriate interventions, fostering clinical judgment [25,26].

When care plans are tied to students’ clinical experiences, they become more meaningful and impactful, further enhancing patient care-related competence [17,25,26]. Through this process, students strengthen their capacity to assimilate and apply knowledge, analyze challenges, and implement solutions, thereby ensuring safer and higher-quality care [3,10,11]. Participants highlighted that care plans empower them to respond more efficiently to patients using evidence-based actions, serving as a clinical guide for designing safe, competent, and patient-centered care.

The increasing complexity of high-quality healthcare services has heightened the workload for healthcare professionals, particularly nurses. In busy clinical environments, the task of writing and maintaining well-prepared care plans often becomes burdensome [28]. The stress of daily practice can lead to care plan writing being neglected, adversely impacting students’ learning and clinical performance [17,18]. Moreover, students may encounter challenges in developing care plans for patients with intrinsic needs, as classroom training typically focuses on specific diseases rather than the multifaceted conditions commonly observed in practice [4,17,19].

The development of critical thinking and problem-solving skills emerged as a recurring theme among participants, highlighting the value of active engagement in the learning process. However, the pivotal role of nurse tutors is equally crucial. Tutors help students recognize the importance of evidence-based care plan writing and provide feedback that encourages self-reflection, enhances communication, and promotes higher-quality learning [29,30,31]. This feedback is vital for pinpointing areas of improvement, ultimately bolstering students’ confidence and competence in delivering safe and effective patient care [29,31]. Participants also indicated that tutors’ clinical experiences serve as invaluable guidance, enriching students’ learning experience and inspiring them to excel [27,28].

To tackle these challenges, students must proactively take the initiative to enhance their skills through self-study and practical learning, ensuring they can create valid, effective care plans for safer patient care [27,29,31]. It is important to acknowledge that the transition from classroom learning to clinical practice in care plan writing is a critical learning journey. This transition not only equips students with the ability to apply knowledge and skills effectively but also fosters positive learning attitudes and motivation for lifelong professional development [24,27]. Shifting from working with scenario cases to real patients represents a valuable learning opportunity, allowing students to translate textbook knowledge into complex clinical situations. Many students reported feeling unprepared for the intricacies of actual patient cases, having primarily practiced with simplified classroom scenarios focused on single health conditions. This disparity became evident when students encountered patients with multiple comorbidities, struggling to prioritize interventions and adapt standardized approaches to individualized care needs. Therefore, program developers and nurse educators should leverage care plans as a core component of the nursing curriculum to improve students’ competence in improving patient care outcomes, with increasing complexity that mirrors students’ developing clinical competence throughout their nursing education. Incorporating real-life clinical stories and case studies can assist students in formulating more tailored care plans, integrating their evidence-based theoretical knowledge and practical skills. Continuous support and regular feedback sessions with tutors encourage students’ self-reflection and enrich their learning experiences [1,4,8].

## 6. Strengths and Limitations

This study highlighted the value of care plan writing in fostering personal and professional development and enhancing competence for clinical readiness. However, it has several limitations. First, all participants were recruited from a single educational institution, limiting the generalizability of the findings to other settings, as experiences may vary based on institutional requirements. Second, the study did not include the perspectives of nurse educators, whose insights could provide valuable guidance for improving the teaching and application of care plans in nursing education.

## 7. Conclusions

This study identified four major themes from undergraduate nursing students’ experiences with care plan writing, highlighting its significance in enhancing competence and fostering personal and professional development. Care plan writing enables students to integrate and apply theoretical knowledge and practical skills, leading to safer and more effective patient care in clinical practice. These findings underscore the value of care plan writing in improving the quality of healthcare services. To maximize the benefits of care plan writing, nurse tutors should emphasize its importance in nursing curricula and encourage consistent practice, particularly during clinical placements. Providing students with adequate support, timely feedback, and guidance is essential for enhancing their competence. Moreover, fostering positive learning attitudes and motivation among students, along with constructive feedback from tutors, can improve the quality of care they deliver.

Care plan writing is a critical learning tool that bridges the gap between classroom education and clinical practice, equipping students with the skills and confidence needed for high-quality patient care. The findings suggest that through thoughtful curriculum design and adequate support systems, care plans can continue to offer transformative learning experiences that prepare students for the complexities of modern patient care.

## Figures and Tables

**Table 1:** Participants’ demographic characteristics.

Demographics	N (%)
Age	
Range	20–25
Gender	
Male	8
Female	7
Year of study	
First year entry	3
Senior year entry	12
Study year	
2 years	3
3 years	8
>3 years	4
Clinical experience	
1 year	7
2 years	8

**Table 2:** Identified themes and subthemes.

Themes	Subthemes
1. Enhancement and integration of knowledge and skills	1. Utilization of evidence-based knowledge and skills
	2. Advanced skill development
	3. Benefits to patient care
2. Initiative learning attitudes and motivation	
3. Adequate support and feedback from tutors	1. Timely feedback from tutors
	2. Sharing of clinical experience by tutors
	3. Learning in a group
4. Difficulties in transitioning from classroom learning to clinical practice	1. Inadequate time/heavy workload
	2. Handling more complicated cases in clinical practice

## Data Availability

The data presented in this study are available on request from the corresponding author. The data are not publicly available to maintain confidentiality.

## Supplementary Materials

The following supporting information can be downloaded at: https://www.mdpi.com/article/10.3390/nursrep15040134/s1, Table S1: The Consolidated Criteria for Reporting Qualitative Research (COREQ-32); Table S2: A coding tree; Supplementary S1: Interview guide; Figure S1: Identified themes and subthemes.

## Public Involvement Statement

There was no public involvement in any aspect of this research.

## Guidelines and Standards Statement

A complete list of reporting guidelines can be accessed via the equator network: https://www.equator-network.org/.

## References

[1] Ballantyne H. (2020). Using nursing care plans to support effective working. InPractice.

[2] Patalagsa J. (2013). Nursing Care Plan: An Evidence-Based Tool for Learning and Providing High Quality Care.

[3] Hooks R. (2016). Developing nursing care plans. Nurs. Stand..

[4] Lucas P., Jesus É., Almeida S., Araújo B. (2023). Relationship of the nursing practice environment with the quality of care and patients’ safety in primary health care. BMC Nurs..

[5] Kumar R. (2021). Concept mapping in nursing education. IJNS Adv..

[6] Wong F.M.F. (2018). A phenomenological research study: Perspectives of student learning through small group work between undergraduate nursing students and educators. Nurse Educ. Today.

[7] Ielapi N., Costa D., Peluso A., Nobile C., Venditti V., Bevacqua E., Andreucci M., Bracale U.M., Serra R. (2022). Wound Care Self-Efficacy Assessment of Italian Registered Nurses and Wound Care Education in Italian Nursing Education System: A Cross-Sectional Study. Nurs. Rep..

[8] Lee Y., Hirai Y., Sarantou M., Kawaguchi A., Wang J. (2025). The Role and Effectiveness of the Life Map Design Tool in Establishing a Care Plan. Arch. Des. Res..

[9] Aldridge J., Eshun A., Meurier C. (2016). Nursing assessment and care planning. Nursing Practice: Knowledge and Care.

[10] Doenges M., Moorhouse M., Murr A. (2019). Nursing Care Plans: Guidelines for Individualizing Client Care Across the Life Span.

[11] Ballantyne H. (2016). Developing nursing care plans. Nurs. Stand..

[12] Burt J., Roland M., Paddison C., Reeves D., Campbell J., Abel G., Bower P. (2012). Prevalence and benefits of care plans and care planning for people with long-term conditions in England. J. Health Serv. Res. Policy.

[13] Ajani K., Moez S. (2011). Gap between knowledge and practice in nursing. Procedia Soc. Behav. Sci..

[14] Tong A., Sainsbury P., Craig J. (2007). Consolidated criteria for reporting qualitative research (COREQ): A 32-item checklist for interviews and focus groups. Int. J. Qual. Health Care.

[15] Colaizzi P.F., Valle R.S., King M. (1978). Psychological research as a phenomenologist views it. Existential-Phenomenological Alternatives for Psychology.

[16] Streubert H.J., Carpenter D.R. (2011). Qualitative Research in Nursing: Advancing the Humanistic Imperative.

[17] Baraz S., Memarian R., Vanaki Z. (2015). Learning challenges of nursing students in clinical environments: A qualitative study in Iran. J. Educ. Health Promot..

[18] Banamwana G., Mandy A.S. (2015). Evaluation of the use and value of nursing care plans in nursing practice at a referral hospital, Kigali, Rwanda: Nurses’ perspectives. Rwanda, J. Ser. F Med. Health Sci..

[19] Burt J., Rick J., Blakeman T., Protheroe J., Roland M., Bower P. (2014). Care plans and care planning in long-term conditions: A conceptual model. Prim. Health Care Res. Dev..

[20] Vera M. (2024). Nursing care plans. Ultimate guide and list. *Nurselabs*. http://nurselabs.com/nursing-care-plans/.

[21] Wager C. (2013). Case study: A critical reflection of implementing a nursing care plan for two hospitalized patients. UK-Vet: Vet. Nurs..

[22] Toney-Butler T., Thayer J. (2022). Nursing Process. StatPearls.

[23] Can G., Erol O. (2012). Nursing students’ perceptions about nursing care plans: A Turkish perspective. Int. J. Nurs. Pract..

[24] Wong F.M.F. (2025). Fostering caring attributes to improve patient care in nursing through small-group work: Perspectives of students and educators. Nurs. Rep..

[25] Karantzas G.C., Avery M.R., MacFarlane S., Mussap A., Tooley G., Hazelwood Z., Fitness J. (2013). Enhancing critical analysis and problem-solving skills in undergraduate psychology: An evaluation of a collaborative learning and problem-based learning approach. Aust. J. Psychol..

[26] Smith J., Rushton M. (2018). Improving student nurses’ confidence in managing the acutely ill patient. Br. J. Nurs..

[27] Lundberg K.M. (2008). Promoting self-confidence in clinical nursing students. Nurse Educ..

[28] Taberna M., Gil Moncayo F., Jané-Salas E., Antonio M., Arribas L., Vilajosana E., Peralvez Torres E., Mesía R. (2020). The Multidisciplinary Team (MDT) Approach and Quality of Care. Front. Oncol..

[29] Chokwe J.M. (2015). Students’ and tutors’ perceptions of feedback on academic essays in an open and distance learning context. Open Prax..

[30] Clynes M.P., Raftery S.E.C. (2008). Feedback: An essential element of student learning in clinical practice. Nurse Educ. Pract..

[31] Lilly J., Richter M., Rivera-Macias B. (2010). Using feedback to promote learning: Student and tutor perspectives. Pract. Res. High. Educ..

