# Supplementary material for: Care Plan Writing in Nursing Education: Challenges, Competence, and Clinical Preparedness
Source: Nurs Rep. 2025 Apr 16;15(4):134. doi: 10.3390/nursrep15040134 (PMC12030564; doi:10.3390/nursrep15040134)

Table S1. Consolidated criteria for reporting qualitative studies (COREQ): 32-item checklist.

No	Item	Guide questions/description	Responses
Domain 1: Research team and reflexivity			
Personal Characteristics			
1.	Interviewer/facilitator	Which author/s conducted the interview or focus group?	The PI & the research assistant
2.	Credentials	What were the researcher's credentials? E.g. PhD, MD	PI obtained Doctoral Degree
3.	Occupation	What was their occupation at the time of the study?	PI is an Associate Professor.
4.	Gender	Was the researcher male or female?	Female
5.	Experience and training	What experience or training did the researcher have?	PI had training in research methods and experience of conducting a phenomenological study with focus group interviews.
Relationship with participants			
6.	Relationship established	Was a relationship established prior to study commencement?	Teacher and students
7.	Participant knowledge of the interviewer	What did the participants know about the researcher? e.g. personal goals, reasons for doing the research	The participants were explained about the study purposes and their involvement before the commencement of the study
8.	Interviewer characteristics	What characteristics were reported about the interviewer/facilitator?	The interviewer is a nurse educator

		e.g. Bias, assumptions, reasons and interests in the research topic	and involved in teaching in small group work. She concerns about caring attributes of nursing students in their professional development.
Domain 2: study design			
Theoretical framework			
9.	Methodological orientation and Theory	What methodological orientation was stated to underpin the study? e.g. grounded theory, discourse analysis, ethnography, phenomenology, content analysis	Yes. It is a phenomenological study. The Colaizzi's method was used to systematically analyze the transcripts.
Participant selection			
10.	Sampling	How were participants selected? e.g. purposive, convenience, consecutive, snowball	15 students
11.	Method of approach	How were participants approached? e.g. face-to-face, telephone, mail, email	Face-to-face focus group interviews
12.	Sample size	How many participants were in the study?	15 student participants
13.	Non-participation	How many people refused to participate or dropped out? Reasons?	None
Setting			
14.	Setting of data collection	Where was the data collected? e.g. home, clinic, workplace	In the study site

15.	Presence of nonparticipants	Was anyone else present besides the participants and researchers?	No
16.	Description of sample	What are the important characteristics of the sample? e.g. demographic data, date	Yes, the sample characteristics were reported.
Data collection			
17.	Interview guide	Were questions, prompts, guides provided by the authors? Was it pilot tested?	Yes, open-end guided questions were provided by the PI and a rehearsal session was done before interview.
18.	Repeat interviews	Were repeat interviews carried out? If yes, how many?	No. Each group was interviewed once
19.	Audio/visual recording	Did the research use audio or visual recording to collect the data?	Digital audio-recording was used during interviews
20.	Field notes	Were field notes made during and/or after the interview or focus group?	The field notes were taken by the research assistant during focus group interviews.
21.	Duration	What was the duration of the interviews or focus group?	The duration of each focus interview was 45 to 90 minutes.
22.	Data saturation	Was data saturation discussed?	Yes. The interview was stopped when data saturation had been reached.
23.	Transcripts returned	Were transcripts returned to participants for comment and/or correction?	No but the PI performed the member check during the interviews. The transcripts were analyzed by the PI and the research assistant independently. Discussion was done until consensus was reached if discrepancies occurred.

Domain 3: analysis and findings			
Data analysis			
24.	Number of data coders	How many data coders coded the data?	Two, PI and the research assistant
25.	Description of the coding tree	Did authors provide a description of the coding tree?	No.
26.	Derivation of themes	Were themes identified in advance or derived from the data?	Themes were derived from the data.
No	Item	Guide questions/description	Responses
27.	Software	What software, if applicable, was used to manage the data?	No
28.	Participant checking	Did participants provide feedback on the findings?	The reviewer would make conclusion to confirm and validate with the participants if the meaning of statements from the participants were accurate.
Reporting			
29.	Quotations presented	Were participant quotations presented to illustrate the themes / findings? Was each quotation identified? e.g. participant number	Yes
30.	Data and findings consistent	Was there consistency between the data presented and the findings?	Yes
31.	Clarity of major themes	Were major themes clearly presented in the findings?	Yes
32.	Clarity of minor themes	Is there a description of diverse cases or discussion of minor themes?	No

Table S2. Coding tree

**Raw Data (Significant Statement)**	**Formulated Meaning**	**Category**	**Theme**
″When we have a case designed by our tutor, we identify the patient's needs and write a care plan. We apply evidence-based knowledge and practical skills, like pathophysiology and pharmacology, to resolve patient problems.″	Application of evidenced-based knowledge and skills to a case	Knowledge and skills	Enhancement and integration of knowledge and skills
″ Care plan writing was new to me when I started the program. At first, it was difficult, but self-practice repeatedly, it became an achievement, especially when caring for patients in clinical practice ″<br><br>Self-motivation is key. Once we realize the importance of care plans, we push ourselves to learn more and improve	Educator input sharpens clinical reasoning	Learning initiatives	Initiative learning attitudes and motivation
″ Practicing is important, but tutor feedback is essential. Sometimes, I thought my care plan was well-written, but the grade didn't reflect that. Seeking feedback helps identify weaknesses and improve.″	Feedback and support	Support from tutor	Adequate support and feedback from tutors
Writing care plans in clinical practice is difficult. During my placement in a female medical unit, I couldn't find time to write a care plan because bedside care took up my entire shift. Even when I tried, I couldn't focus because I was constantly interrupted.	Obstacles to write care plans during clinical practice	Difficulties from classroom to practice	Difficulties in transition from classroom learning to clinical practice

Supplementary S1: Interview guide

1. Share with me your experience of writing care plan during your study.
2. How do you think of writing care plan?
3. Share with me the usefulness of care plan writing for patient care
4. Share with me the advantages of writing care plan for your learning
5. Share with me the advantages of writing care plan for your clinical practice
6. Share with me the challenges or difficulties of writing care plan in your study
7. What can help you improve your care plan writing?

Figure S1. Identified themes and subthemes

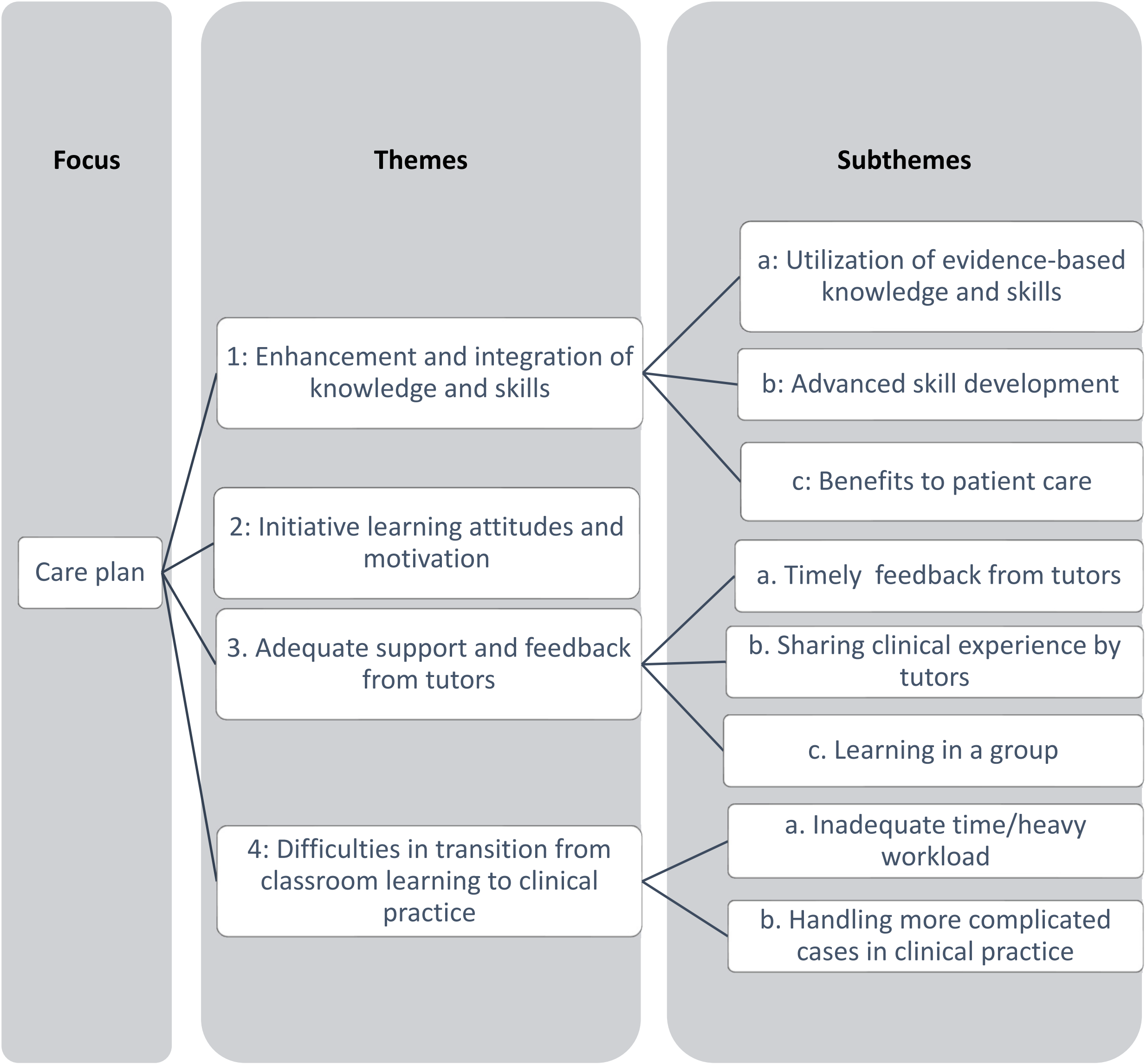

Supplement: Supplementary file 1 [file nursrep-15-00134-s001.zip › nursrep-3546208-supplementary.pdf]
